# Evaluating the Diagnostic Performance of a Multiplex Polymerase Chain Reaction Panel for the Detection of Synovial Fluid Pathogens in the Setting of Periprosthetic Joint Infection

**DOI:** 10.7759/cureus.91300

**Published:** 2025-08-30

**Authors:** John L Miamidian, Krista Toler, Subramaniam Somasundaram, Alex McLaren, Jason Cholewa, Carl Deirmengian

**Affiliations:** 1 Department of Diagnostics Research and Development, Zimmer Biomet, Warsaw, USA; 2 Orthopaedic Surgery, University of Arizona College of Medicine-Phoenix, Phoenix, USA; 3 Orthopaedic Surgery, The Rothman Orthopaedic Institute, Philadelphia, USA; 4 Orthopaedic Surgery, Thomas Jefferson University, Philadelphia, USA

**Keywords:** diagnosis, dna sequencing, pathogen, periprosthetic joint infection, total hip arthroplasty, total knee arthroplasty

## Abstract

Background

This prospective study assessed the performance of a commercial multiplex polymerase chain reaction (PCR) panel in diagnosing periprosthetic joint infection (PJI).

Methods

Synovial fluid samples from hip and knee arthroplasties, submitted to a clinical diagnostic laboratory for PJI workup, were tested with multiplex PCR to identify a specific panel of organisms. Clinical biomarker test results and microbiological culture were used to classify samples as 'not infected', 'inconclusive', and 'infected' using a modified 2018 International Consensus Meeting (ICM) criteria for PJI. The purpose of this study was to evaluate the specificity, the on- and off-panel sensitivity, and the clinical utility of the multiplex PCR panel for diagnosing PJI.

Results

The multiplex PCR panel demonstrated 100% specificity (55/55 samples; 95% CI: 94%-100%) with no false-positive cases. Sensitivity was 96% (95% CI: 89%-99%) for the infected samples (85/89 samples), yielding microorganisms included as targets in the PCR panel. For all culture-positive samples classified as infected, including off-panel organisms, the sensitivity of the multiplex PCR panel for pathogen identification was 58% (87/150 samples; 95% CI: 50%-66%). The panel identified microorganisms in 18% (9/50) of the culture-negative samples classified as infected and in 2% (1/50) of culture-negative samples classified as inconclusive.

Conclusion

The multiplex PCR panel assessed in this study demonstrated excellent specificity, with a negligible false-positive rate, yet still identified organisms in the setting of culture-negative infection. Sensitivity for organisms included on the multiplex panel was also excellent. Clinicians must be aware that the panel will yield negative results in the setting of organisms which are not on the panel, such as *Staphylococcus epidermidis*.

## Introduction

The clinical recognition of culture-negative periprosthetic joint infection (PJI) has created a need for alternative microorganism detection methods [1]. Polymerase chain reaction (PCR), first applied to synovial fluid (SF) about two decades ago, was hoped to be a tool that could replace traditional culturing techniques for identifying pathogens in SF [2]. However, molecular methods based on genetic amplification have not become standard practice due to the difficulty of maintaining high sensitivity while minimizing false positives in microorganism detection [3,4].

Initial applications of PCR demonstrated its capacity to amplify genetic material from specific microorganisms in SF [5]. This led to efforts targeting the amplification of universal DNA regions in pathogens, using technologies like next-generation sequencing and broad-range 16S PCR, to identify any potential pathogens present [4,6]. Despite these advancements, such efforts frequently yield clinical results characterized by high false-positive rates and the identification of numerous microorganisms within each positive sample [7-11]. These limitations have impeded widespread clinical adoption of amplification-based methodologies in the diagnosis and management of PJI.

A multiplex PCR panel has been recently authorized to identify PJI pathogens in SF [12]. Its main advantage is targeted detection of pathogens associated with PJI, thereby reducing false positives from contaminant amplification. However, the main drawback is the inability to detect microorganisms not targeted by the panel [13]. Nevertheless, the multiplex PCR panel represents a unique approach in applying molecular techniques to SF. The purpose of this study was to evaluate the specificity, the on- and off-panel sensitivity, and the clinical utility of the multiplex PCR panel for PJI pathogen identification. This work was previously presented as an abstract and poster at the American Academy of Orthopaedic Surgeons (AAOS) 2024 Annual Meeting held in San Francisco from February 12-16, 2024. 

## Materials and methods

Sample qualification and storage

The study involved an analysis of hip and knee SF samples collected from patients undergoing routine care and submitted to a single laboratory for comprehensive PJI testing. The time required to collect the 305 study samples extended from March 30 to June 20, 2023. Previously de-identified remnant samples and data were utilized in this study in accordance with the Institutional Review Board approval (WIRB-Copernicus Group Institutional Review Board; approval no. 20150222). 

Inclusion criteria were: 1) specimen from hip or knee; 2) sample received within one day of aspiration; 3) sufficient remnant sample volume (500 µL) to obtain a valid test according to the manufacturer's recommendations; 4) sample meeting internal laboratory specimen integrity requirements (i.e. red blood cell count ≤1 million cells/µl and absorbance at 280 nm wavelength within the acceptable range of 0.342 to 1.190) [14]; and 5) biomarker results available for SF C-reactive protein (SF-CRP), alpha-defensin (AD), white blood cell count (SF-WBC), neutrophil percentage (SF-PMN%), and culture. 

Samples were transported at ambient temperature via overnight courier for clinical diagnostic testing. Transport time was limited to one day due to the unknown effect of ambient conditions on sample stability, though the manufacturer's instructions recommend refrigerated storage for up to seven days. Qualifying remnant samples were refrigerated for up to six days, then frozen at -80°C until multiplex PCR testing. Source joint, basic demographics, and clinical SF testing results were recorded, and frozen samples were thawed and tested using the multiplex PCR panel. The time required to test the 305 study samples extended from May 3 to June 28, 2023.

SF biomarker and multiplex PCR panel testing

Comprehensive SF testing was performed by a Clinical Laboratory Improvement Amendments (CLIA)-certified laboratory specializing in SF analysis (CD Laboratories, Zimmer Biomet, Towson, MD). Microbiological culture screening used anaerobic and aerobic bottles monitored for up to seven days with the BACT/ALERT system (BioMerieux, Salt Lake City, UT). Positive bottles underwent further analysis via the plate-streak method for microorganism isolation and the Vitek II system (BioMerieux, Durham, NC) for identification and antimicrobial susceptibility testing (AST). In cases involving fungal organisms or when in-house identification failed, samples were sent to a reference lab (Quest, Chantilly, VA) for further identification and susceptibility testing.

Multiplex PCR analysis was performed at CD Laboratories using the BioFire Joint Infection Panel (BioFire Diagnostics, Salt Lake City, UT) following the manufacturer's instructions. Each specimen, requiring about 200 µL, was processed using a disposable pouch and the BioFilm Array instrument, which automates nucleic acid extraction, reverse transcription, amplification, and result analysis. The pouches were provided in-kind, and the instrument was loaned by the manufacturer for the study. The PCR panel detects 15 gram-positive bacteria (seven anaerobes, 11 species-specific, four species groups/genus), 14 gram-negative bacteria (one anaerobe, nine species-specific, five species groups/genus), two yeast results (one species-specific, one genus), eight antimicrobial resistance genes (ARGs) from SF specimens within about an hour per sample.

Sample classification

A total of 305 samples were tested of which 279/305 (91.5%) were from knees and 26/305 (8.5%) were from hips. Patients were 68±10 years of age and were predominantly male patients (59%; 180/305). 

A PJI classification was applied to each sample based on the modified 2018 International Consensus Meeting (ICM) criteria [15] using SF-CRP in place of serum CRP. SF-CRP has been demonstrated to be as good or better than serum CRP as a biomarker for PJI diagnosis [16-18]. The composite score comprised of three points for either SF-WBC >3000 cells/µL or AD Signal to Cutoff ≥1, two points for SF-PMN% >70, two points for SF-CRP >6.6 mg/L [19], and two points for a single positive SF culture. A score <3 was classified as 'not infected', >5 was classified as 'infected', and 3-5 as 'inconclusive' (Table 1). 

**Table 1:** Modified 2018 International Consensus Meeting definition of PJI, as applied in this study PJI: Periprosthetic joint infection; WBC: white blood cell count; AD: alpha-defensin; CRP: C-Reactive Protein; PMN (%): polymorphonuclear neutrophil (neutrophil percentage); N/A: Not applicable.

Criteria	Threshold	Score	Decision
Synovial WBCs (cells/µL) OR	3000	3	>5 infected
				AD Signal to Cutoff	1	3-5 inconclusive
Synovial CRP (mg/L)	6.6		2			
Synovial PMN (%)	70	2	<3 not infected
Single positive culture	N/A	2	

Scoring of the modified 2018 ICM definition included preoperative SF data but did not include serum data or postoperative data.

The qualifying samples were enrolled consecutively into four target categories, with a sample size predetermined for each. Cohorts of 50 provide reasonably narrow confidence intervals (CIs) for assessment of point estimates with upper and lower confidence bounds. We collected 55 negative control samples considering the potential for false positives, so that at least 50 samples were negative by PCR panel within that cohort. We also collected 150 positive control samples, considering the prevalence of on-target organisms so that at least 50 samples were positive by the PCR panel within the cohort. We applied the same minimum cohort size, collecting 50 samples for each of two additional cohorts that were culture negative but not ICM negative. Once the required samples for a category were collected, further collection for that category was discontinued. The first category, designed to be a negative control group, was characterized by a 'not infected' classification and a negative culture result (n=55). The second category, designed to be a positive control group, was characterized by an 'infected' classification and a positive culture result (n=150). Two additional cohorts, consisting of (1) culture-negative 'infected' (n=50) and (2) culture-negative 'inconclusive' (n=50) samples were also included. Samples classified as 'not infected' with positive culture results, samples classified as 'inconclusive' with a score of three, and samples classified as 'inconclusive' with a score of four or five with positive culture results were excluded from the four study cohorts.

Data analysis

Descriptive statistics were generated for each study cohort using mean ± standard deviation (SD). The multiplex PCR panel’s diagnostic sensitivity and specificity were calculated using the positive (infected/culture-positive) and negative (not infected/culture-negative) control groups, respectively. Performance metrics were reported with 95% CIs using the Clopper-Pearson method, except in some cases, where Score Confidence intervals were used to match the PCR manufacturer's methodology. Sensitivity was assessed for the entire positive control cohort and non-target PCR microorganisms. Additional analysis included evaluating the clinical utility of microorganism identification by multiplex PCR, particularly for the culture-negative inconclusive cohort (4/5), where accurate identification would confirm infection. Cross-reactivity of the multiplex PCR with non-PCR targets, based on the culture-identified microorganisms in the culture-positive infected cohort, was also analyzed. Finally, ARGs detected by the PCR panel were evaluated in relation to reference AST results. Analyses were conducted using Microsoft Excel (Microsoft Corp., Redmond, WA, US) and Minitab version 18.1 (Pennsylvania, USA).

## Results

Characteristics of cohorts

SF biomarker characterization revealed low concentrations in the negative-control cohort, high concentrations in the positive-control cohort, and moderate-high concentrations in the clinical utility cohorts (Table 2).

**Table 2:** Overall results of the comprehensive synovial fluid (SF) testing, in terms of host-response biomarkers Data reported as mean ± standard deviation; WBC: white blood cell; AD: alpha-defensin; CRP: C-Reactive Protein; PMN%: percentage of neutrophils.

Biomarker	Negative control	Positive control	Inconclusive (4/5)	Infected
	Not infected	Infected		
	Culture-negative	Culture-positive	Culture-negative	Culture-negative
	(n=55)	(n=150)	(n=50)	(n=50)
AD (signal to cutoff)	0.12 ± 0.10	3.14 ± 1.22	1.12 ± 1.06	1.77 ± 1.40
SF-CRP (mg/L)	1.49 ± 2.30	37.52 ± 32.94	5.90 ±5.52	33.64 ± 26.79
SF-WBC cells/µL	752 ± 692	57962 ± 65418	12534 ± 29255	23635 ± 23351
SF-PMN%	33.7 ± 15.1	92.69 ± 5.70	79.1 ± 10.47	89.12 ± 7.42

Microorganism profile in the positive control cohort

**Figure 1:**
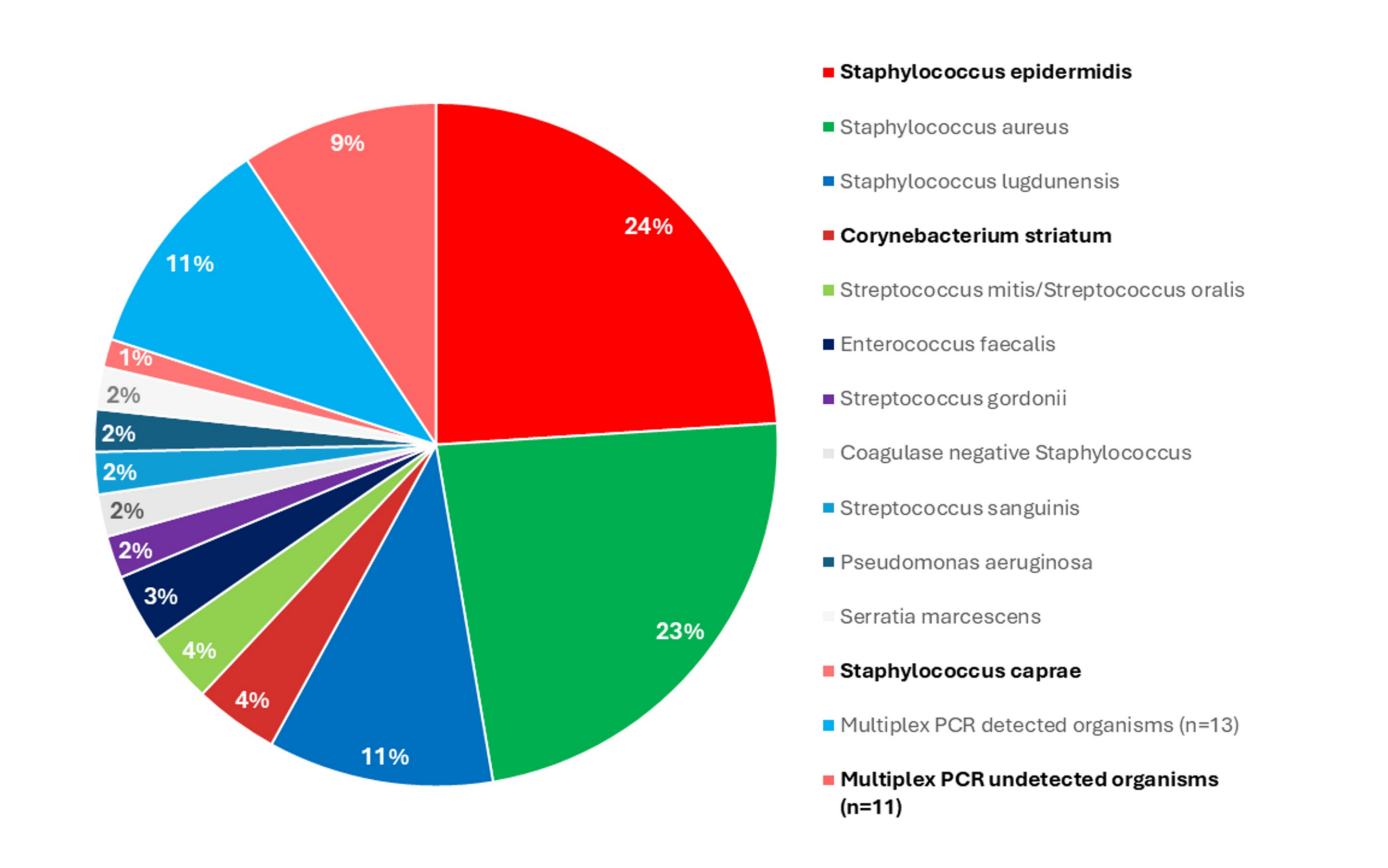
Overall occurrence of microorganisms in the study The 12 most common microorganisms identified by culture are included in the legend. The microorganisms marked in bold (red sections of the pie chart) indicate those not included in the multiplex polymerase chain reaction (PCR) panel. Three samples were identified by culture as 'coagulase-negative staphylococcus' and could not be more definitively identified.

The 150 sample positive control (infected and culture-positive) cohort included 59% (89/150) of samples culturing positive for microorganisms included as targets in the multiplex PCR panel, 38% (57/150) which cultured positive for microorganisms not targeted by the multiplex PCR panel, and 3% (4/150) of samples that were culture positive without definitive identification (i.e., coagulase-negative staphylococcus (n=3) and organism most closely resembling *Staphylococcus schleiferi* (n=1)). *Staphylococcus epidermidis* was the leading off-target cultured organism at a frequency of 24% (36/150), followed by *Corynebacterium striatum* at a frequency of 4% (6/150) (Figure 1).

Multiplex PCR panel performance

There were no false-positive PCR panel results among the negative-control samples (not infected/culture-negative), yielding a specificity of 100% (55/55; 95% CI: 94%-100%). 

The PCR panel correctly identified 85/89 target microorganisms (96% on-panel target sensitivity; 95% CI: 89%-99%). Two *Staphylococcus aureus, *one *Streptococcus sanguinis, *and one *Streptococcus mitis/oralis *were not detected by the multiplex PCR panel, and no other identification was made by the PCR panel in these particular samples. For the overall positive control cohort (n=150) including on-panel and off-panel microorganisms, the sensitivity was 58% (87/150; 95% CI: 50%-66%). Specificity and sensitivity, per microorganism, for the multiplex PCR panel ranged from 89.5 to 100% (Table 3).

**Table 3:** Multiplex polymerase chain reaction organism-level sensitivity and specificity (with 95% score CIs) TP: True positive; TN: True negative; FP: False positive; FN: False megative ^*^Finegoldia magna was detected in an infected sample with a positive culture for *S. epidermidis* and as a second organism along with *S. aureus *in an infected sample with a positive culture for *S. aureus*.  Specificity was based solely on not infected, culture-negative samples. ^**^Streptococcus agalactiae was detected by multiplex PCR as a second organism along with *S. aureus* in an infected sample with a positive culture for *S. aureus*.  Specificity was based solely on not infected, culture-negative samples.

Analyte	Sensitivity			Specificity		
							TP/(TP + FN)	%	95% CI	TN/(TN + FP)	%	95% CI
	Gram Positive Bacteria						
Anaerococcus prevotii/vaginalis	0/0	-	-	55/55	100	93.5-100%
Clostridium perfringens	0/0	-	-	55/55	100	93.5-100%
Cutibacterium avidum/granulosum	0/0	-	-	55/55	100	93.5-100%
Enterococcus faecalis	5/5	100	56.6-100%	55/55	100	93.5-100%
Enterococcus faecium	0/0	-	-	55/55	100	93.5-100%
Finegoldia magna*	0/0	-	-	55/55	100	93.5-100%
Parvimonas micra	2/2	100	34.2-100%	55/55	100	93.5-100%
Peptoniphilus	0/0	-	-	55/55	100	93.5-100%
Peptostreptococcus anaerobius	0/0	-	-	55/55	100	93.5-100%
Staphylococcus aureus	33/35	94.3	81.4-98.4%	55/55	100	93.5-100%
Staphylococcus lugdunensis	16/16	100	80.6-100%	55/55	100	93.5-100%
Streptococcus spp.	17/19	89.5	68.6-97.1%	55/55	100	93.5-100%
Streptococcus agalactiae**	2/2	100	34.2-100%	55/55	100	93.5-100%
Streptococcus pneumoniae	0/0	-	-	55/55	100	93.5-100%
Streptococcus pyogenes	0/0	-	-	55/55	100	93.5-100%
Gram Negative Bacteria						
Bacteroides fragilis	0/0	-	-	55/55	100	93.5-100%
Citrobacter	0/0	-	-	55/55	100	93.5-100%
Enterobacter cloacae complex	1/1	100	20.7-100%	55/55	100	93.5-100%
Escherichia coli	1/1	100	20.7-100%	55/55	100	93.5-100%
Haemophilus influenzae	0/0	-	-	55/55	100	93.5-100%
Kingella kingae	0/0	-	-	55/55	100	93.5-100%
Klebsiella aerogenes	0/0	-	-	55/55	100	93.5-100%
Klebsiella pneumoniae group	1/1	100	20.7-100%	55/55	100	93.5-100%
Morganella morganii	0/0	-	-	55/55	100	93.5-100%
Neisseria gonorrhoeae	0/0	-	-	55/55	100	93.5-100%
Proteus spp.	1/1	100	20.7-100%	55/55	100	93.5-100%
Pseudomonas aeruginosa	3/3	100	43.8-100%	55/55	100	93.5-100%
Salmonella spp.	0/0	-	-	55/55	100	93.5-100%
Serratia marcescens	3/3	100	43.8-100%	55/55	100	93.5-100%
Yeast						
Candida	2/2	100	34.2-100%	55/55	100	93.5-100%
Candida albicans	0/0	-	-	55/55	100	93.5-100%

Minimal non-target cross-reactivity was observed, with only one discordant sample between the multiplex PCR panel (*Finegoldia magna*) and culture (*Staphylococcus epidermidis*). Two samples had a second microorganism detected by the multiplex PCR panel (sample 1: *Streptococcus agalactiae*; sample 2: *Finegoldia magna*) in addition to the *Staphylococcus aureus* which was concordant between multiplex PCR and culture. 

Clinical utility of the multiplex PCR panel

For the first clinical utility cohort of the infected culture-negative samples, the multiplex PCR panel identified microorganisms in 9/50 samples (18%; 95% CI: 8%-31%) (Table 4). 

**Table 4:** Microorganisms detected by multiplex polymerase chain reaction (PCR) in the infected, culture-negative cohort A (+) indicates the value exceeded the modified 2018 International Consensus Meeting definition of PJI, while a (-) indicates the value was below the threshold. ^1^All samples had an infection classification score of 7. SF: Synovial fluid; CRP: C-reactive protein; AD: alpha defensin; WBC: white blood cell; PMN%: neutrophil percentage.

Microorganism	Joint^1^	SF-CRP (mg/L)	SF-AD (Signal to cut-off)	SF-WBC (cells/µL)	SF-PMN%
Streptococcus agalactiae	Right knee	16 (+)	2.284 (+)	5472 (+)	94.4 (+)
Streptococcus spp.	Right knee	79.1 (+)	1.201 (+)	40893 (+)	95.9 (+)
Haemophilus influenzae	Left knee	15.5 (+)	2.355 (+)	7740 (+)	87.7 (+)
Klebsiella aerogenes	Left knee	27.4 (+)	1.199 (+)	2511 (-)	89.7 (+)
Enterococcus faecalis	Right knee	22.4 (+)	3.121 (+)	62017 (+)	98.8 (+)
Staphylococcus aureus	Right knee	71.8 (+)	3.873 (+)	47385 (+)	94.1 (+)
Streptococcus agalactiae	Right hip	21 (+)	3.307 (+)	23393 (+)	96.5 (+)
Proteus spp.	Right knee	14.8 (+)	4.275 (+)	13315 (+)	97.1 (+)
Staphylococcus aureus	Right knee	11 (+)	4.662 (+)	2809 (-)	96.6 (+)

For the second clinical utility cohort of inconclusive culture-negative samples, the multiplex PCR panel identified microorganisms in 2% (1/50; 95% CI: 0%-11%) of samples (*Staphylococcus aureus*). 

Further analysis of the infected samples which yielded positive cultures also revealed some clinical utility from the multiplex PCR analysis. There were four samples which had non-definitive culture results including three “*coagulase-negative staphylococcus*” and one sample sent to a reference lab for further evaluation, which returned a result of “organism most closely resembles *Staphylococcus schleiferi*.” Multiplex PCR identified one of the three “*coagulase-negative staphylococcus*” and the “organism most closely resembles *Staphylococcus schleiferi*” as *S. lugdunensis*.

Detection of antimicrobial resistance genes (ARG)

This analysis was limited to multiplex PCR panel ARG targets within the culture system of AST. Overall, eight of 10 samples with a reference antimicrobial indicating resistance in the culture system were detected as having ARGs by PCR. This included eight of the nine methicillin-resistant *S. aureus* (MRSA) and zero of the one cefotaximase (CTX-M) resistance gene on an *Enterobacter cloacae complex*-positive sample with AST results indicating resistance to ceftriaxone, amikacin, aztreonam, and piperacillin/tazobactam (Table 5).

**Table 5:** Summary of the detection of antimicrobial resistance by the multiplex polymerase chain reaction (PCR) compared to automated, abbreviated doubling dilution technique AST is presented as resistant (R) with minimum inhibitory concentration (MIC); ARG: antimicrobial-resistance gene; mecA: methicillin resistance gene A; mecC: methicillin resistance gene C; MRSA: methicillin-resistant staphylococcus aureus; MREJ: Mec right extremity junction; AST: antimicrobial susceptibility testing; R: resistant.

Microorganism identified by culture	Multiplex PCR ARG	Relevant comments about the culture	Ceftriaxone (AST)	Oxacillin (AST)
Enterobacter cloacae complex	None detected	None	R, >=64	Not Available; resistance to oxacillin was not tested for this microorganism
Staphylococcus aureus	mecA/C and MREJ (MRSA)	This isolate is confirmed to be MRSA	No result	R, >=4
Staphylococcus aureus	mecA/C and MREJ (MRSA)	This isolate is confirmed to be MRSA	No result	R, >=4
Staphylococcus aureus	mecA/C and MREJ (MRSA)	This isolate is confirmed to be MRSA	No result	R, >=4
Staphylococcus aureus	mecA/C and MREJ (MRSA)	This isolate is confirmed to be MRSA	No result	R, >=4
Staphylococcus aureus	mecA/C and MREJ (MRSA)	This isolate is confirmed to be MRSA	No result	R, >=4
Staphylococcus aureus	None detected	This isolate is confirmed to be MRSA	No result	R, >=4
Staphylococcus aureus	mecA/C and MREJ (MRSA)	This isolate is confirmed to be MRSA	No result	R, >=4
Staphylococcus aureus	mecA/C and MREJ (MRSA)	This isolate is confirmed to be MRSA	No result	R, >=4
Staphylococcus aureus	mecA/C and MREJ (MRSA)	This isolate is confirmed to be MRSA	No result	R, >=4

Neither the microorganism nor the ARG was detected by multiplex PCR for the one discordant MRSA sample.

## Discussion

This study prospectively assessed the diagnostic performance of a commercial multiplex PCR panel in detecting SF pathogens from patients with suspected PJI. The panel had 100% specificity and 96% sensitivity for on-panel microorganisms, but sensitivity dropped to 58% when off-panel microorganisms were included. The panel provided clinical utility by identifying microorganisms in 18% of culture-negative infections and detecting ARGs. Additionally, it offered rapid results within an hour, faster than traditional cultures, which can take at least 48 hours or even weeks to identify microorganisms and perform AST. Retaining the panel’s current specificity while adding *Staphylococcus epidermidis* and *Corynebacterium* ​​​*striatum*, which were cultured in 24% and 4% of the infected samples, respectively, could improve its clinical utility and routine use for the identification of organisms in PJI.

The current study of 305 SF samples represents an extensive formal diagnostic evaluation of the BioFire multiplex PCR platform for joint infection. Azad et al. [20] utilized 60 archived SF samples diagnosed using the criteria from the Infectious Disease Society of America (IDSA), demonstrating 91% (95% CI: 73 to 98%) sensitivity and 100% specificity for on-panel microorganisms. However, like the current study, the sensitivity decreased to 56% (95% CI: 41 to 70%) when including off-panel microorganisms. Due to the absence of antimicrobial-resistant microorganisms, they were unable to evaluate the multiplex PCR’s performance in detecting ARGs. Schoenmakers et al. [21] also conducted a study evaluating the BioFire multiplex PCR platform, including a total of 45 SF samples from suspected native infections and PJI. They found a sensitivity of 80.6% (95% CI: 64-91%) for on-panel microorganisms and a specificity of 100%. Again, they found that the inclusion of samples with off-panel microorganisms decreased the sensitivity to 61.3%, aligning with the current study and Azad et al. Other authors have reported sensitivities between 90.5% to 100% and specificities of 93% to 100% in a combination of native septic arthritis and suspected PJI cases [13,22,23]. Therefore, all six studies evaluating the BioFire multiplex PCR platform identified high sensitivity in identifying on-panel microorganisms, decreased sensitivity when including samples with off-panel microorganisms, and near-perfect specificity with exceedingly few false positive results in any of the studies.

Many previous studies on this multiplex PCR platform lacked enough samples to assess ARG detection or system performance in ambiguous cases. This study included 10 samples where AST identified antimicrobial-resistant microorganisms. One sample contained ceftriaxone-resistant *Enterobacter cloacae complex*, which the multiplex PCR did not detect. AST revealed this microorganism was also resistant to amikacin, aztreonam, and piperacillin/tazobactam. It is possible the genetic mechanism of resistance is not by the CTX-M pathway. Conversely, Esteban et al. [22] reported the detection of CTX-M in five out of five samples. Of the nine remaining samples with resistant organisms in this study, all yielded MRSA by AST, with 89% of cases detected by the multiplex-PCR, which is comparable to the high level of detection reported by Esteban et al. [22]. The high rate of antimicrobial resistance detection is clinically important, as it would facilitate early initiation of effective treatment in a population of patients with a concerning diagnosis [24,25]. That being said, many genetic resistance mechanisms to antibiotics are unknown from testing results of the PCR panel and this multiplex PCR panel cannot replace traditional culture with AST results.

This study also explored aspects of clinical utility not addressed in smaller studies. Among the 50 culture-negative infections, the multiplex PCR panel identified microorganisms in 18% of cases, confirming PJI and providing a target for antimicrobial treatment. Given its low false-positive rate, detecting microorganisms in culture-negative PJI is credible. Detection rates could further increase with the inclusion of *Staphylococcus epidermidis* and *Corynebacterium striatum*. Additionally, this study found that only one out of the 50 samples that were inconclusive and culture-negative yielded a microorganism detected by multiplex PCR. Given the reasonably high sensitivity of this multiplex PCR platform and the relatively high sensitivity of traditional cultures, we believe that many of these inconclusive samples are actually not infected, but rather represent another diagnosis presenting with low-grade inflammation.

Overall, this study in combination with previous studies [13,20,22,23] demonstrates multiplex PCR to be a reliable technology that can provide high sensitivity while maintaining a negligible false positive rate. With the addition of *Staphylococcus epidermidis* and *Corynebacterium striatum*, we believe that this technology could become a routinely useful assay for evaluating SF samples from patients with suspected PJI. In its present form, this PCR panel could be implemented in cases where PJI is strongly suspected but the pathogen has not yet been identified. This could enable faster microorganism identification or identification of a microorganism in the setting of culture negative infection, thereby guiding treatment from empirical to targeted antibiotic therapy in some patients. The main disadvantage of multiplex PCR is that it targets specific bacteria, but this also results in a very low false positive rate due to the high specificity of targeted amplification. In contrast, next-generation sequencing can detect any bacteria but has a 10-30% false positive rate [4,9,26], rendering clinical interpretation very difficult. 

Limitations of this study include the lack of serology or tissue cultures and limited demographic and clinical history data due to the use of a specialized laboratory. Therefore, the study is limited to the sensitivity and specificity of the PCR panel compared to the SF components of the ICM definition. Nonetheless, the authors of the 2018 ICM definition of PJI noted that “Not all tests are needed to use this proposed definition, and a preoperative diagnosis can be made without the need for intraoperative findings” [15]. Additionally, since this was a secondary analysis of SF samples submitted for routine testing, we couldn't control the sample delivery conditions. Instead of being refrigerated as per the manufacturer’s instructions, samples were transported overnight at ambient temperature. However, we minimized this effect by selecting samples received within one day of aspiration, and the high sensitivity and specificity observed suggest the transport had minimal impact on the performance of multiplex PCR panel. This study also included a majority of samples from the knees as compared to the hips. While we did not control for this difference in terms of sample size, we included both of these joints as the pathogen profiles are similar with similar coverage of the PCR panel. The diagnostic criteria are also not different for the two sites [15]. The results, however, would not apply to shoulders, where *Cutibacterium acnes* is the most prevalent organism identified in infections, and this organism is not detected by the PCR panel.

## Conclusions

The multiplex PCR joint infection panel tested in this study demonstrated 58% sensitivity and 100% specificity for the identification of pathogens in SF samples undergoing PJI testing. The limited number of panel targets, notably the exclusion of *Staphylococcus epidermidis*, reduces the maximum achievable sensitivity. The multiplex PCR panel assessed in this study demonstrated excellent sensitivity and specificity for the on-panel identification of pathogens in the SF samples from patients undergoing comprehensive PJI testing. However, negative results in the clinical setting should be interpreted with the understanding that some common organisms, such as *Staphylococcus epidermidis* and *Corynebacterium striatum,* are not detected by the panel. As a platform technology, the multiplex PCR panel provides a near-ideal combination of high sensitivity with low false-positive results. Because this performance profile is limited by the pathogens included as panel targets, the technology could be optimally leveraged with an expanded microorganism detection profile.
